# Effects on Proliferation and Differentiation of Human Umbilical Cord-Derived Mesenchymal Stem Cells Engineered to Express Neurotrophic Factors

**DOI:** 10.1155/2016/1801340

**Published:** 2015-11-15

**Authors:** Yi Wang, Youguo Ying, Xiaoyan Cui

**Affiliations:** ^1^Department of Orthopaedics, Tongren Hospital, Shanghai Jiao Tong University School of Medicine, Shanghai 200336, China; ^2^Department of Intensive Care Unit, Shanghai 9th People's Hospital, Shanghai Jiao Tong University School of Medicine, Shanghai 201999, China; ^3^Stem Cell Research Center, Tongji University School of Medicine, Shanghai 200092, China

## Abstract

Mesenchymal stem cells (MSCs) are multipotential cells with capability to form colonies *in vitro* and differentiate into distinctive end-stage cell types. Although MSCs secrete many cytokines, the efficacy can be improved through combination with neurotrophic factors (NTFs). Moreover, MSCs are excellent opportunities for local delivery of NTFs into injured tissues. The aim of this present study is to evaluate the effects of overexpressing NTFs on proliferation and differentiation of human umbilical cord-derived mesenchymal stem cells (HUMSCs). Overexpressing NTFs had no effect on cell proliferation. Overexpressing NT-3, BDNF, and NGF also had no significant effect on the differentiation of HUMSCs. Overexpressing NTFs all promoted the neurite outgrowth of embryonic chick E9 dorsal root ganglion (DRG). The gene expression profiles of the control and NT-3- and BDNF-modified HUMSCs were compared using RNA sequencing and biological processes and activities were revealed. This study provides novel information about the effects of overexpressing NTFs on HUMSCs and insight into the choice of optimal NTFs for combined cell and gene therapy.

## 1. Introduction

Mesenchymal stem cells (MSCs) are multipotential cells with capability to form colonies* in vitro *and differentiate into distinctive end-stage cell types, such as adipocytes, osteoblasts, and chondrocytes, as well as other connective tissues and neuronal cells [1–4]. They are also a heterogeneous population and can be isolated from several tissues, including bone marrow, adipose, umbilical cord blood, umbilical cord, and amnion. Human umbilical cord-derived mesenchymal stem cells (HUMSCs) have no ethical concerns and are capable of being isolated and expanded easily* in vitro*; therefore, they are a promising source of MSCs.

MSCs have been used to animal models and clinical trials for treatment of many diseases, such as myocardial infarcts, graft-versus-host disease, stroke, and spinal cord injury [5]. MSCs possess many properties directed to the hurdle for disease treatment, such as paracrine effect, immunomodulatory effect, anti-inflammatory effect, antiapoptotic effect [6, 7]. They can migrate and secrete a variety of cytokines in an injury environment, including insulin-like growth factor (IGF), brain-derived neurotrophic factor (BDNF), vascular endothelial growth factor (VEGF), granulocyte-macrophage colony stimulating factor (GM-CSF), fibroblast growth factor- (FGF-) 2, and transforming growth factor (TGF) [8].

Although MSCs secrete many cytokines, the efficacy can be improved through combination with neurotrophic factors (NTFs) [9]. NTF alone does not cross the blood-brain barrier and is difficultly delivered to the central nervous system [10]. Moreover, MSCs are excellent opportunities for local delivery of NTFs into injured tissues. Multifunctional therapies seem to be extremely promising because they counteract multiple disease mechanisms and combine both neuroprotective and neuroregenerative agents [11]. Genetic modification of MSCs with neurotrophic factors can not only increase secretion of peptide or total length of protein with potential to repair injury of central nervous system, but also promote the survival of themselves and the survival or regeneration of neurons [12]. In this context, it is essential to evaluate the effects of overexpressing NTFs on MSCs, including neurotrophin 3 (NT-3), BDNF, glial cell line-derived neurotrophic factor (GDNF), and nerve growth factor (NGF).

## 2. Materials and Methods

### 2.1. Cell Isolation, Culture, and Phenotype Identification

HUMSCs were isolated as previously described [13]. Ten human umbilical cords were obtained after the delivery of normal-term babies with institutional review board approval. A portion of the umbilical cord was cut into approximately a 7 cm long segment. The segment was then placed immediately into 25 mL of DMEM/F12 (Gibco) supplemented with 10% fetal bovine serum (FBS; Gibco) and antibiotics (100 U/mL penicillin, 100 *μ*g/mL streptomycin). The tubes were brought to the laboratory on the ice for dissection within 4 hours. Umbilical cord segment was washed three times with phosphate-buffered saline without calcium and magnesium (PBS; Gibco) and dissected longitudinally utilizing aseptic technique. The umbilical vein and both umbilical arteries were removed. The umbilical cord segment was cut into 0.5–1 mm^3^ tissue block and incubated in 3 mL of 0.2% Collagenase Type II at 37°C for 1 hour. Fivefold volume of complete medium was added and the supernatant was filtered with 70 *μ*m filter after free settling for 20 minutes. Cells were collected with centrifugation at 1500 rpm for 5 minutes and finally plated in plastic culture flasks at a concentration of 5 × 10^3^/cm^2^. After 3 days, nonadherent cells were removed by washing three times with PBS. Medium was changed every 2-3 days. HUMSCs were identified antigen expression using flow cytometry with human MSC analysis kit (BD Biosciences) containing CD90 FITC/CD105 PerCP-Cy5.5/CD73 APC.

### 2.2. Lentiviral Vectors and MSC Transduction

MSCs were, respectively, transduced with pLVX-IRES-ZsGreen1 Vector with the insertion of the full length cDNA of NT-3, BDNF, GDNF, and NGF, or without insertion (as control). MSCs at a concentration of 1 × 10^4^/cm^2^ were infected with lentivirus for 72 hours. The volume of lentivirus used for each transduction was determined by titration as the required volume to generate 90%–95% ZsGreen1 positive MSCs after 3 days.

### 2.3. RNA Extraction and Real-Time Polymerase Chain Reaction

Total RNA was extracted with TRIzol (Invitrogen), following the manufacturer's instructions. Genomic DNA contamination in RNA samples was removed and reverse transcription using 1 *μ*g of RNA was performed using QuantiTect Reverse Transcription Kit (Qiagen). For all mRNAs detected, SYBR* Premix Ex Taq* II (Perfect Real Time) (Takara) was used for real-time polymerase chain reaction, using primers listed in Table 1.

### 2.4. Measurement of NTF Levels in Cell Culture Supernatant

Transduced MSCs were plated in six-well plates (5,000 cells per square centimeter). After overnight, medium was changed to 2 mL per well of DMEM/F12 and incubated for 48 hours. Then, supernatants were collected to confirm overexpression and secretion of each factor using human enzyme-linked immunosorbent assay (ELISA) kit following the manufacturer's instructions (Abcam). Cell number was determined for normalization.

### 2.5. Western Blots

For detection of overexpressing NT-3, BDNF, GDNF, and NGF in HUMSCs, proteins in transduced cells were extracted using RIPA (radio immunoprecipitation assay) Lysis Buffer supplemented with 1 mM of PMSF (Beyotime Institute of Biotechnology). Proteins were loaded in 12% SDS-PAGE gels and transferred to PVDF membranes (Millipore). After blocking for 1 hour, membranes were incubated with primary antibodies (diluted at 1 : 200) overnight at 4°C. Antibodies against NT-3, BDNF, GDNF, and NGF were purchased from Santa Cruz Biotechnology.

### 2.6. Cell Proliferation Assay

Transduced MSCs were plated in triplicate in 96-well plates at a density of 2,000 per well and then the live cell count was assayed using the Cell Counting Kit-8 (CCK-8) (Dojindo, Kumamoto, Japan) according to the manufacturer's protocol. In brief, 10 *μ*L of CCK-8 solution was added to each well and the samples were incubated for 3 h. The absorbance was measured at 450 nm.

### 2.7. Differentiation Procedures

The adipogenic, osteogenic, and chondrogenic differentiation potentials of HUMSCs were determined by incubating them in differentiation media from the Human Mesenchymal Stem Cell Functional Identification Kit (SC006; R&D Systems, UK).

#### 2.7.1. Adipogenic Differentiation Protocol

HUMSCs were seeded onto 12 mm coverslips at a density of 3.7 × 10^4^ cells per well in *α*-MEM basal medium supplemented with 10% FBS + 100 U/mL penicillin, 100 *μ*g/mL streptomycin, and 2 mM L-glutamine. Cells were cultured in a 37°C and 5% CO_2_ incubator. When 100% confluence was reached, the medium was replaced with the Adipogenic Differentiation Medium (R&D Systems). The medium was replaced every 3 days. After 2 weeks, cells seeded on coverslips were washed with PBS twice and fixed with 4% formaldehyde (Beijing Solarbio Science & Technology) for 20 min at room temperature. For Oil Red O staining, cells were washed once with PBS and stained for 30 minutes with Oil Red O (Sigma) and for 5 minutes with DAPI (4′, 6-diamidino-2-phenylindole; 1 : 1,000, Sigma). After washing twice with PBS, cells were photographed under a microscope (Nikon).

#### 2.7.2. Osteogenic Differentiation

For osteogenic induction, 7.4 × 10^3^ HUMSCs per well were cultured in *α*-MEM basal medium supplemented with 10% FBS + 100 U/mL penicillin, 100 *μ*g/mL streptomycin, and 2 mM L-glutamine. After 50–70% confluency, the medium was replaced with the Osteogenic Differentiation Medium (R&D Systems), with a medium change every 3 days. After culture for 21 days, cells were fixed with 4% paraformaldehyde for 20 minutes, washed three times with PBS, and stained with mouse anti-human osteocalcin antibody and NorthernLights 557 Fluorochrome-conjugated donkey anti-mouse secondary antibody (R&D Systems). Cells from 3 wells of each cell line were photographed with a fluorescence microscope. All images of osteogenic and chondrogenic differentiation were analyzed using ImageJ (NIH; Bethesda, MD, USA).

#### 2.7.3. Chondrogenic Differentiation

For chondrogenic differentiation, 2.5 × 10^5^ cells were centrifuged at 200 ×g for 5 minutes at room temperature and resuspended with DMEM/F-12 basal medium supplemented with ITS Supplement (R&D Systems) and 100 U/mL penicillin, 100 *μ*g/mL streptomycin, and 2 mM L-glutamine. The cells were centrifuged again at 200 ×g for 5 minutes, resuspended in Chondrogenic Differentiation Medium (R&D Systems), and centrifuged. Pellets were incubated at 37°C and 5% CO_2_ for 21 days, with medium change every 3 days. The pellets were fixed with 4% paraformaldehyde in PBS for 20 minutes at room temperature and frozen and sectioned using standard cryosectioning methods. The sections were stained with the goat anti-human aggrecan antibody and NorthernLights 557 Fluorochrome-conjugated donkey anti-goat secondary antibody (R&D Systems). Cells from triplicate of each cell line were photographed with a fluorescence microscope.

### 2.8. Neurite Outgrowth of Embryonic Chick Dorsal Root Ganglion (DRG)

Fertilized eggs were purchased from the stock farm of Shanghai Academy of Agricultural Sciences. The eggs were incubated under 37.6°C, 60% humidity for 9 days. DRG was dissected cleanly from E9 chick embryos and cultured in six-well tissue culture plates (2 wells for each testing sample) with five DRGs in each well. Excess media were drained off and 920 *μ*L of ice-cold fresh-made BME-collagen mixture was added into each well to cover entire surface. One mL of each testing sample (1 : 1 diluted with 10% FBS-DMEM/F12) was added into the wells, respectively, on the top of collagen. Serum-free DMEM/F12 (1 : 1 diluted with 10% FBS-DMEM/F12) was used as blank and NT-3 (final concentration: 3.5 ng/mL) and NGF (final concentration: 10 ng/mL) were used as positive controls. The plates were incubated at 37°C with 5% CO_2_ for 24 hours and the ganglions were examined using an Olympus inverted microscope. The photo of each ganglion was divided into 8 sectors and the longest neurites in each sector were joined to form an octagon. The area of this octagon (*A*_tot_) and the area of the DRG (*A*_DRG_) were obtained using ImageJ software (v 1.4.3.). The neurite outgrowth index (OI) was calculated by the formulation OI = (*A*_tot_/*π*)^1/2^ − (*A*_DRG_/*π*)^1/2^.

### 2.9. RNA Sequencing and Analysis

To compare gene expression profiles among control and NT-3- and BDNF-overexpressing HUMSCs, these cells were submitted to RNA isolation using TRIzol Reagent (Invitrogen), and then total RNA was digested with DNase I (Invitrogen) to ensure that samples were not contaminated with genomic DNA. Six samples derived from 2 allogeneic cell lines with three different modifications of control, NT-3, and BDNF were performed. Library preparation and paired end sequencing (length of 101 bp) were done using Illumina HiSeq 2500 sequencer. Short reads were aligned to the reference human genome (Homo_sapiens GRCh37) using TopHat. Gene annotations were from Ensembl packaged into iGenome (Illumina). The sequence reads were used to calculate overall gene expression in terms of RPKM (reads per kilobase of exon per million mapped reads). The differentially expressed transcripts were screened using the cufflinks procedure. Genes with gene expression greater than or equal to 2-fold (*P* ≤ 0.01 compared with other groups) were identified in control and NT-3- and BDNF-overexpressing HUMSCs, respectively. Functional enrichment analyses were performed.

Genes related to cytokine-cytokine receptor interactions, which had RPKM values of more than 5 in control and NT-3- and BDNF-modified HUMSCs, were selected based on the KEGG PATHWAY Database (http://www.genome.jp/kegg/pathway.html).

### 2.10. Data Presentation and Statistical Analysis

All values in figures represent averages with the standard error of mean as error bars. All significant differences were evaluated using ANOVA or repeated measures of general linear model, comparing raw data (not normalized) of conditions with control. Significance level was set at *P* < 0.05.

## 3. Results

### 3.1. Overexpression of NT-3, BDNF, GDNF, and NGF in HUMSCs

**Figure 1:**
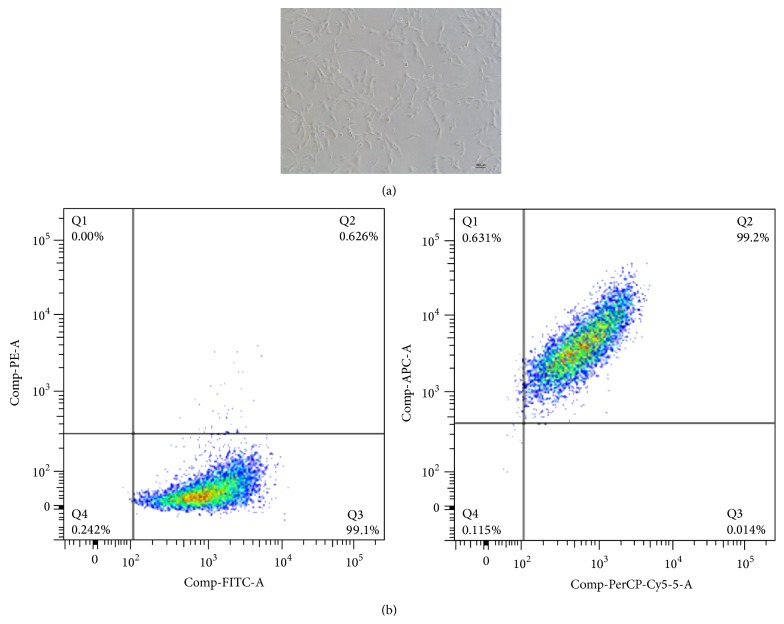
Phenotype identification of HUMSCs using flow cytometry. (a) Morphology of HUMSCs. Scale bar = 100 *μ*m. (b) There are 98.3% of HUMSCs, positive for CD90 FITC/CD105 PerCP-Cy5.5/CD73 APC but negative for CD34, CD45, CD11b or CD14, CD19 or CD79*α*, and HLA-DR.

**Figure 2:**
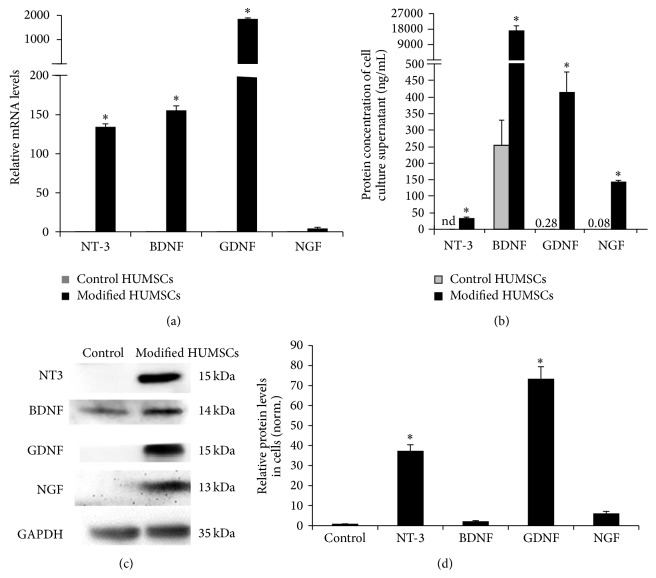
HUMSCs were transduced with control lentiviral vectors or those designed to overexpress NTFs. Overexpression of NTFs was then confirmed at both mRNA and protein levels. (a) mRNA was extracted from HUMSCs 3 days after transduction and measured by real-time reverse transcription polymerase chain reaction (*n* = 3). (b) Protein levels of NTF were measured in supernatant of HUMSCs using enzyme-linked immunosorbent assay (*n* = 3). (c) Protein levels of NTF measured in HUMSCs using western blot. (d) Semiquantitative analysis of results of western blot. ^*∗*^ *P* < 0.05 versus control (*n* = 3).

HUMSCs were similar to fibroblasts (Figure 1(a)). There were 98.3% of HUMSCs positive for CD90 FITC, CD105 PerCP-Cy5.5, and CD73 APC but negative for CD34, CD45, CD11b or CD14, CD19 or CD79*α*, and HLA-DR (Figure 1(b)). After transduction of HUMSCs with NT-3, BDNF, GDNF, and NGF lentivirus, we confirmed that production and secretion of these NTFs were increased in HUMSCs. As shown in Figure 2(a), the mRNA of each of NT-3, BDNF, and GDNF was significantly increased and the mRNA of NGF was slightly increased in HUMSCs transduced with the respective NTFs using real-time polymerase chain reaction. Each NTF was also found to be significantly increased in HUMSC culture supernatant upon overexpression using ELISA (Figure 2(b)). In HUMSCs transduced with the respective NTFs using western blots, the protein of each of NT-3 and GDNF was significantly increased and the protein of each of BDNF and NGF was slightly increased (Figures 2(c) and 2(d)).

### 3.2. Overexpressing NTFs Had No Effect on Cell Proliferation

**Figure 3:**
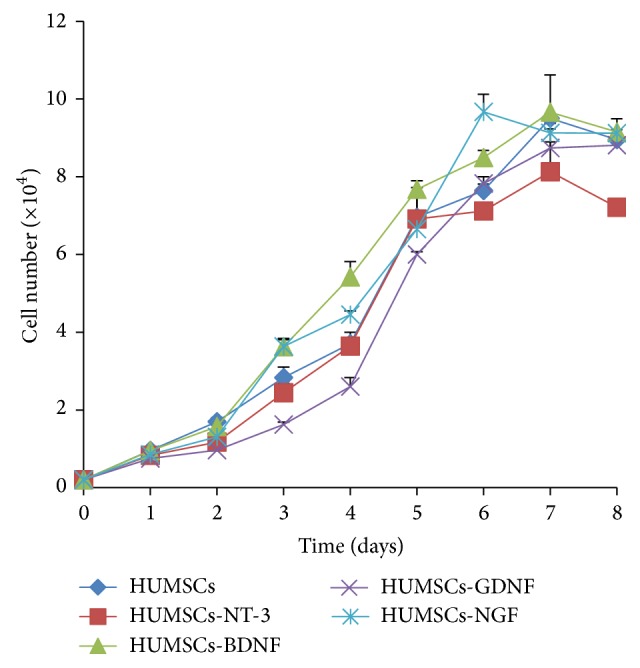
Cell growth curve of control and modified HUMSCs (P_5_, *n* = 3).

Viable cell count assay was performed for cell proliferation of transduced HUMSCs. As shown in Figure 3, overexpression of NTFs did not significantly affect HUMSC growth compared with control HUMSCs.

### 3.3. Overexpressing NTFs Had No Effect on the Adipogenic Differentiation of HUMSCs

**Figure 4:**
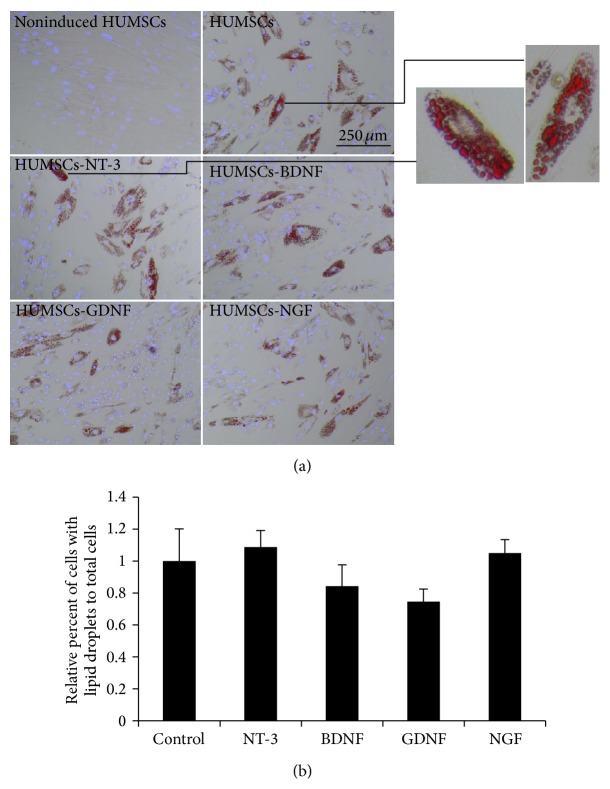
The effect of overexpression of NTFs on the adipogenic differentiation of HUMSCs. Transduced MSCs were cultured in adipogenic induction medium for 14 days. (a) Cells stained with Oil Red O and pictured in representative areas. Scale bar = 250 *μ*m. (b) Relative percentage of adipocytes to total cells stained with DAPI (*n* = 6).

The adipogenic differentiation potential from each NTF-overexpressing HUMSC population was determined using microscopic count of adipocyte-like cells based on oil droplet accumulation. After culturing MSCs with adipogenic induction medium for 14 days, cells with large lipid droplets were observed (Figure 4(a)). Overexpression of BDNF and GDNF led to only a minor, but nonsignificant, reduction in the adipogenic differentiation (Figure 4(b)).

### 3.4. Osteogenic Differentiation of HUMSCs Is Inhibited by Overexpression of GDNF

**Figure 5:**
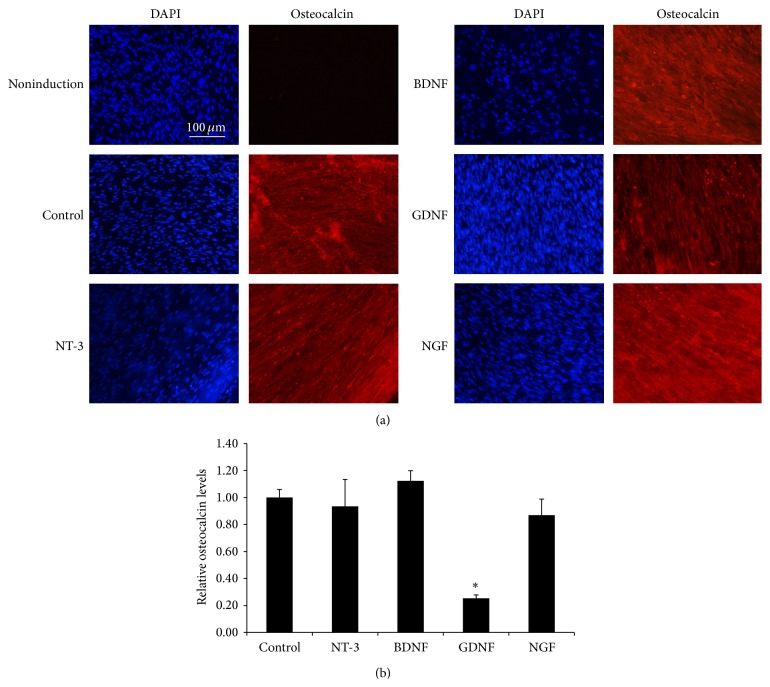
The effect of overexpression of NTFs on the osteogenic differentiation of MSCs. Transduced MSCs were cultured in osteogenic induction medium for 21 days. (a) Cells were stained with anti-human osteocalcin antibody and pictured in representative areas. Scale bar = 100 *μ*m. (b) Relative osteocalcin levels. ^*∗*^ *P* < 0.05 versus control (*n* = 6).

The effects of overexpressing NTFs on the osteogenic differentiation potential of HUMSCs were evaluated by culturing transduced cells for 21 days in Osteogenic Differentiation Medium and then staining cells with anti-human osteocalcin antibody (Figure 5(a)). Osteocalcin level in HUMSCs engineered to overexpress GDNF was significantly lower under standard culture conditions (Figure 5(b)).

### 3.5. Overexpressing NTFs Had No Effect on the Chondrogenic Differentiation of HUMSCs

**Figure 6:**
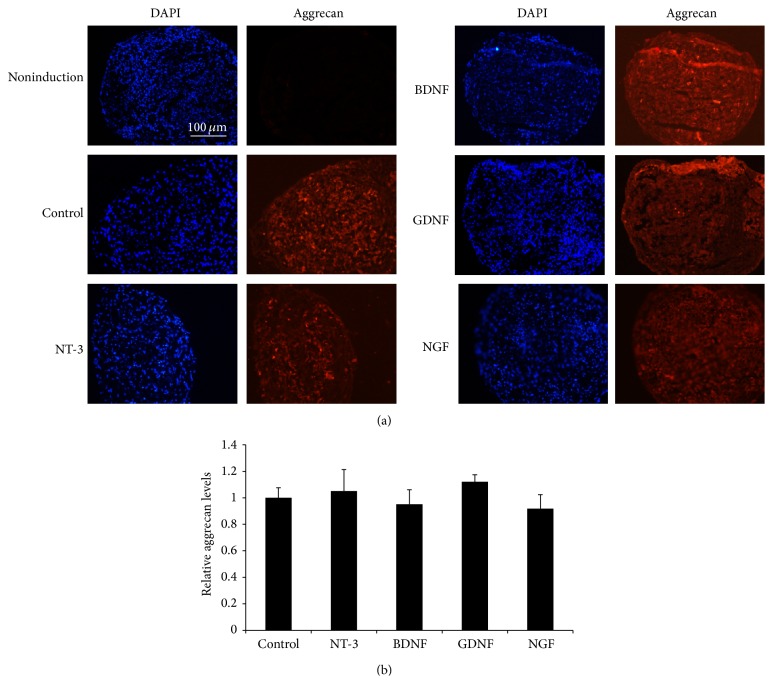
The effect of the overexpression of NTFs on the chondrogenic differentiation of MSCs. Transduced MSCs were cultured in chondrogenic induction medium for 21 days. (a) Cells were stained with anti-human aggrecan antibody and pictured in representative areas. Scale bar = 100 *μ*m. (b) Relative aggrecan levels (*n* = 6).

The effects of overexpressing NTFs on the chondrogenic differentiation potential of HUMSCs were evaluated by culturing transduced cells for 21 days in Chondrogenic Differentiation Medium and then staining cells with anti-human aggrecan antibody (Figure 6(a)). Aggrecan level in HUMSCs engineered to overexpress GDNF was slightly higher and aggrecan level in HUMSCs engineered to overexpress BDNF and NGF was slightly lower under standard culture conditions (Figure 6(b)).

### 3.6. Overexpression of NTFs from HUMSCs Promoted the Neurite Outgrowth of Embryonic Chick E9 DRG

**Figure 7:**
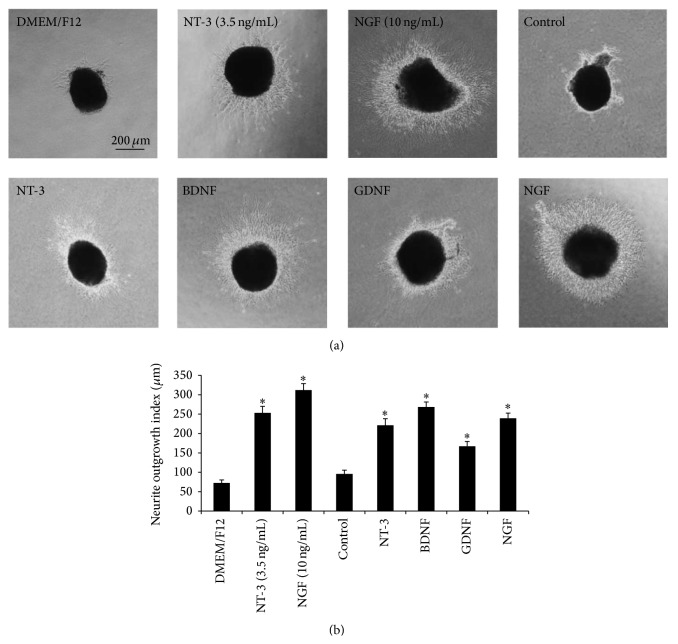
Effects of overexpression of NTFs from HUMSCs on the neurite outgrowth of embryonic chick DRG. (a) Representative images of E9 DRG culture for 24 hours (scale bar = 200 *μ*m). (b) DRG cultured in blank culture medium extended short neurites. NT-3 (3.5 ng/mL), NGF (10 ng/mL), and overexpression of NTFs from HUMSCs treatment effectively promoted neurite outgrowth. Neurite extension of DRG treated with cell culture supernatant of HUMSCs (control) was similar to that of blank group. ^*∗*^ *P* < 0.05 versus blank or control (*n* = 10).

Short neurite extension was observed in DRG cultured in DMEM-F12 (blank, 1 : 1 diluted with 10% FBS-DMEM), while obvious neurite outgrowth was noted in DRG cultured with overexpression of NTFs from HUMSCs (1 : 1 diluted with 10% FBS-DMEM), 3.5 ng/mL NT-3 or 10 ng/mL NGF (Figure 7(a)). Neurite outgrowth was assessed in at least ten E9 chick DRGs for each sample. The outgrowth index of overexpression of NTFs from HUMSCs, 3.5 ng/mL NT-3 or 10 ng/mL NGF treated groups, was significantly increased compared with blank group (Figure 7(b), ^*∗*^ *P* < 0.05), while the cell culture supernatant of control HUMSCs did not exhibit any promotive effect.

### 3.7. Gene Expression Data Analysis

**Figure 8:**
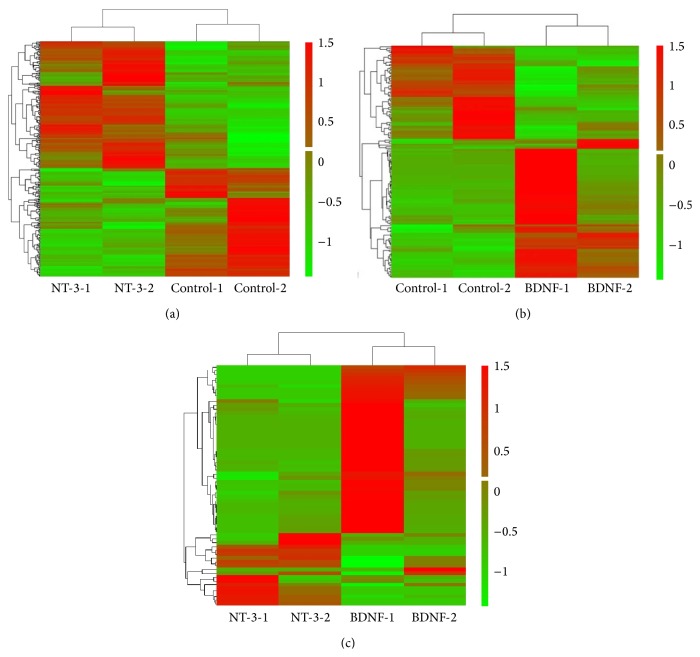
Heat map of the differentially expressed gene set between the different genetic modifications. (a) NT-3 modification and control. (b) BDNF modification and control. (c) NT-3 and BDNF modification.

Six samples derived from 2 allogeneic cell lines (−1 and −2) with three different modifications of control, NT-3, and BDNF were performed. The heat map of the differentially expressed gene set is shown in Figure 8 between the different genetic modifications. There were 317 and 144 genes differentially expressed (greater than or equal to 2-fold) between NT-3-modified and control HUMSCs and between BDNF-modified and control HUMSCs, respectively (Figures 8(a) and 8(b)). The most significant biological processes were “circulatory system development” and “locomotion” for NT-3- and BDNF-modified HUMSCs compared with control, respectively. There were 63 genes differentially expressed (greater than or equal to 2-fold) between NT-3- and BDNF-modified HUMSCs (Figure 8(c)), and the most significant biological processes were “circulatory system development.”

**Figure 9:**
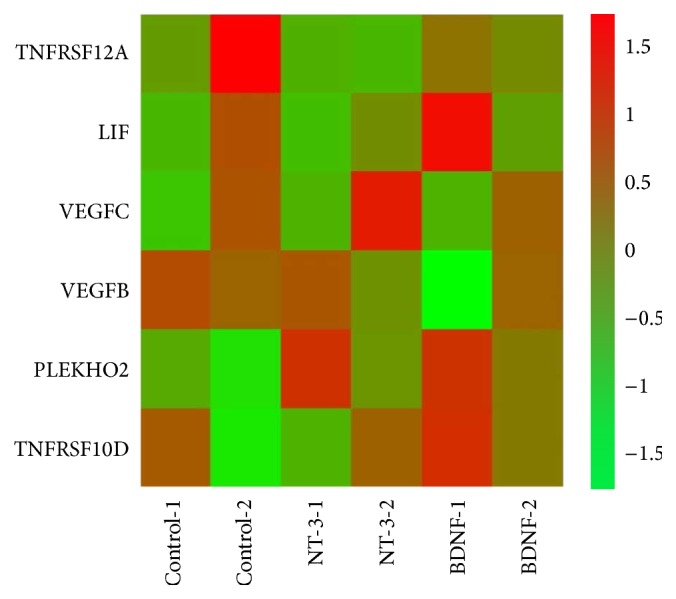
Heat map showing genes related to cytokine-cytokine receptor interaction with RPKM values of more than 5 in three groups.

Gene expression related to cytokine-cytokine receptor interactions among groups was analyzed to clarify the molecular basis of the heterogeneity. HUMSCs have been shown to display anti-inflammatory and immunomodulatory properties, and we also focused on comparative analysis of genes related to cytokine-cytokine receptor interactions in control and NT-3- and BDNF-modified HUMSCs. There were 6 genes with RPKM values of more than 5 related to cytokine-cytokine receptor interactions. Clustering highlighted two inflammatory related genes VEGFC and PLEKHO2 with higher expression in NT-3-modified HUMSCs. Clustering also highlighted three genes LIF, PLEKHO2, and TNFRSF10D with higher expression in BDNF-modified HUMSCs, respectively (Figure 9).

## 4. Discussion

MSCs can secrete a variety of NTFs and exert their functions partly through paracrine effect. Human MSCs derived from dental pulp (hDPSCs), bone marrow (hBMSCs), and adipose (hAMSCs) secreted multiple neuroprotective NTFs that promoted the growth of neurites [14]. To further enhance the efficacy of HUMSCs, we conducted genetic modification of HUMSCs. The effects of various NTFs' overexpression on MSCs* in vitro *are rarely studied in a comparative manner. First, we examined the levels of NTF overexpression in HUMSCs after transduction with the respective lentiviral vectors. The mRNA levels and protein secretion of NT-3, BDNF, and GDNF were significantly increased compared with those of the control. Although the mRNA level of overexpressing NGF was slightly increased compared with the control, the protein level of NGF overexpressed in HUMSCs culture supernatant was significantly increased. Therefore, all four overexpressed mature NTFs can be released from cells and perform their functions. The protein level of BDNF in modified HUMSCs measured using western blot was rather low compared with NT-3 and GDNF. This protein reduction of BDNF in cells may be due to excess release into cell culture supernatant. We also noted significant effects on the neurite outgrowth of the MSCs engineered to express NT-3, BDNF, GDNF, and NGF, indicating that these NTFs were biologically active.

Overexpression of NTFs did not significantly affect HUMSC growth compared with control HUMSCs. In our experimental setting, overexpression of GDNF inhibited the osteogenic differentiation of HUMSCs. GDNF might induce the downregulation of gene specific for osteogenic differentiation and more work is needed to study the mechanism of reduced osteogenic differentiation. GDNF is necessary for normal neuromuscular development and exists in embryonic limb and muscle at high levels at the time of innervation [15]. GDNF can increase neural sprouting and prevent cell death [16–18]. Overexpression of NT-3, BDNF, and NGF had no significant effects on adipogenic, osteogenic, and chondrogenic differentiation of HUMSCs. These data suggest that MSCs engineered to overexpress NT-3, BDNF, and NGF in a controlled manner might be a better candidate for alleviating nervous system disease. Other investigators demonstrated that BDNF overexpressing human umbilical cord blood-MSCs (MSCs-BDNF) yielded an increased number of neuron-like cells and MSCs-BDNF exhibited decreased labeling for MSCs-related antigens such as CD73 and CD90 and decreased potential to differentiate into mesodermal lineages [19]. The differences between our experimental results and their results may be due to the different cell lines used and different experimental setting. We used umbilical cord jelly-derived MSCs and they used umbilical cord blood-derived MSCs.

RNA sequencing analyses can provide new insight into the variable biological properties among control and modified HUMSCs. We performed RNA sequencing analyses for control and NT-3- and BDNF-modified HUMSCs because we are more interested in NT-3 and BDNF. Compared with control HUMSCs, the most significant biological processes were “circulatory system development” and “locomotion” for NT-3- and BDNF-modified HUMSCs, respectively. Gene analyses provide some cues for MSC-mediated anti-inflammatory and immunomodulatory properties of different groups. The anti-inflammatory and immunoregulation-related gene VEGFC (vasculogenesis and angiogenesis) and PLEKHO2 (involved STAT3 pathway) were expressed highly in NT-3-modified HUMSCs. Three genes PLEKHO2, leukemia inhibitory factor (LIF, cytokine activity and receptor binding, induction of neuronal cell differentiation, and immune tolerance at the maternal-fetal interface) [20, 21], and TNFRSF10D (playing an inhibitory role in TRAIL-induced cell apoptosis) [22] were expressed highly in BDNF-modified HUMSCs. VEGFC activated lymphatic vessels in the skin lead not only to an expanded lymphatic network with enhanced fluid drainage, but also to a potent inhibition of acute and chronic skin inflammation [23]. In a model of chronic inflammatory arthritis, VEGFC increased lymphangiogenesis and lymphatic flow and also reduced the severity of joint lesions [24]. The antagonist of the LIF receptor increased gene transcripts for the proinflammatory cytokines tumor necrosis factor, interleukin-1*β*, and interleukin-6 during the inflammatory phase [25]. Hence, LIF can regulate the inflammatory response. Moreover, LIF regulated nonclassical* MHC* class I gene expression and has been suggested to contribute to the localized immunosuppressive environment in endometrium [26]. Therefore, genetic modification with NT-3 and BDNF can also upregulate anti-inflammatory and immunoregulation-related genes in HUMSCs.

In conclusion, NT-3 and BDNF modifications had no effect on proliferation and differentiation of HUMSCs and may be excellent opportunities for combined cell and gene therapy. However, extensive efficacy (including neuronal differentiation) and safety experiments need to be done before MSCs/NTFs therapy could ever be considered.

## Figures and Tables

**Table 1:** Primers used for real-time polymerase chain reaction.

Gene	Forward primer	Reverse primer
GAPDH	GCACCGTCAAGGCTGAGAAC	TGGTGAAGACGCCAGTGGA
NT-3	CATTCGGGGACACCAGGTC	TTTGCACTGAGAGTTCCAGTGTTT
BDNF	GAACTCCCAGTGCCGAACTACC	TTATGAATCGCCAGCCAATTCTC
GDNF	TGCAGTCTTTGCCTAACAGCAAT	GCCACGACATCCCATAACTTCAT
NGF	ATGCTGGACCCAAGCTCA	TGATCAGAGTGTAGAACAACATGGA
